# First-language raters’ opinions when validating word recordings for a newly developed speech reception threshold test

**DOI:** 10.4102/sajcd.v65i1.555

**Published:** 2018-03-29

**Authors:** Seema Panday, Harsha Kathard, Mershen Pillay, Wayne Wilson

**Affiliations:** 1 Discipline of Audiology, School of Health Sciences, University of KwaZulu-Natal, South Africa; 2 Department of Health and Rehabilitation Sciences, University of Cape Town, South Africa; 3 Discipline of Speech Language Therapy, School of Health Sciences, University of KwaZulu-Natal, South Africa; 4 School of Health and Rehabilitation Sciences, University of Queensland, Australia

## Abstract

**Background:**

The purpose of this study was to consider the value of adding first-language speaker ratings to the process of validating word recordings for use in a new speech reception threshold (SRT) test in audiology. Previous studies had identified 28 word recordings as being suitable for use in a new SRT test. These word recordings had been shown to satisfy the linguistic criteria of familiarity, phonetic dissimilarity and tone, and the psychometric criterion of homogeneity of audibility.

**Objectives:**

The aim of the study was to consider the value of adding first-language speakers’ ratings when validating word recordings for a new SRT test.

**Method:**

A single observation, cross-sectional design was used to collect and analyse quantitative data in this study. Eleven first-language isiZulu speakers, purposively selected, were asked to rate each of the word recordings for pitch, clarity, naturalness, speech rate and quality on a 5-point Likert scale. The percent agreement and Friedman test were used for analysis.

**Results:**

More than 20% of these 11 participants rated the three-word recordings below ‘strongly agree’ in the category of pitch or tone, and one-word recording below ‘strongly agree’ in the categories of pitch or tone, clarity or articulation and naturalness or dialect.

**Conclusion:**

The first-language speaker ratings proved to be a valuable addition to the process of selecting word recordings for use in a new SRT test. In particular, these ratings identified potentially problematic word recordings in the new SRT test that had been missed by the previously and more commonly used linguistic and psychometric selection criteria.

## Introduction

Speech reception threshold (SRT) testing is routinely used as part of the basic audiological assessment of hearing (Ramkissoon, Proctor, Lansing & Bilger, 2002). It is typically conducted by presenting a series of words to a listener who must repeat those words as heard. This can be performed, using headphones, or in the free-field, depending on the age and abilities of the listener. The SRT score is the presentation level (typically reported in decibels hearing level [dB HL]) required for a listener to correctly repeat 50% of a list of presented words. This score is used to quantify the listener’s hearing level for speech and to cross-check the listener’s pure-tone audiometry thresholds (the gold standard test for hearing thresholds) (Gelfand, 2001).

Two factors are commonly considered when selecting the words to be included in an SRT test for use in clinical practice: linguistic familiarity and homogeneity of audibility. Linguistic familiarity considers the linguistic properties of words in the language of persons for whom the SRT test is being developed. It identifies the need to choose words most likely to be familiar to most first-language speakers of that language (Kruger & Mazor, 1987). Homogeneity of audibility refers to the ‘ease at which a word is understood when spoken at a constant level of intensity’ (Silman & Silverman, 1991, p. 61). It considers several psychoacoustic properties including each word’s psychometric function and prosodic pattern when spoken (Nissen, Harris, Jennings, Eggert & Buck, 2005). A word’s psychometric function is described as its percentage of correct identification by presentation level (Kruger & Mazor, 1987; Liu & Shi, 2013; MacPherson & Akeroyd, 2014). These functions show that increasing the presentation level of a word increases the probability that a listener will repeat it correctly. The slope of a word’s psychometric function should be steep to provide for more precise estimations of SRT (Hudgins, Hawkins, Karlin & Stevens, 1947) and greater sensitivity to changes in a listener’s ability to understand speech. A word’s prosodic pattern refers to several features including its length, accent, stress, tone and intonation (Fox, 2000). These features carry different meanings in different languages and are often determined by measuring the pitch and energy contours of the word (Panday, Kathard, Pillay & Govender, 2009).

Although linguistic criteria and homogeneity of audibility are clearly important in the development of SRT tests in audiology, these factors alone may not be sufficient to ensure that the final SRT test adequately represents all facets of the construct it has been designed to measure (Maxwell & Satake, 2006). In the context of an SRT test, this refers to the need to satisfy not only linguistic and psychoacoustic criteria, such as those discussed above but also broader criteria, such as the appropriateness of the word recordings for the communities in which the SRT test will be used. This can be done by including expert speakers of the language from the communities in which the test is recorded. These persons can provide immediate and direct feedback as to the appropriateness of the proposed word recordings, being trialled by rating factors such as word intonation, tone and clarity, and representation in terms of dialect. Such ratings could contribute to the final SRT test’s content and ecological validity (Theunissen, Swanepoel & Hanekom, 2009), where content validity refers to how well the test words represent the content domain the test was designed to measure, and ecological validity refers to how the test words represent everyday word use in the communities in which the test will be used (Theunissen et al., 2009).

This study reflects on the lessons learnt during the development of a new SRT test for use with speakers of isiZulu in KwaZulu-Natal (KZN), South Africa (Panday, Kathard, Pillay & Govender, 2007, 2009). In particular, it considers the value of adding expert speaker ratings of test word recordings where those expert speakers were drawn from the population in which the test is to be used.

## Methods

### Aim

The aim of the study was to consider the value of adding first-language speakers’ ratings when validating word recordings for a new SRT test.

### Research design

A single observation, cross-sectional design (Maxwell & Satake, 2006) was used to collect and analyse quantitative data in this study.

### Participants

The participants were 11 first-language isiZulu speakers (3 men and 8 women, aged 18–60 years with a mean age of 36 years) purposively selected for measuring the suitability of the SRT test word recordings. These participants were permanent residents of KZN Province in South Africa and were self-reported native speakers of isiZulu with normal hearing thresholds (≤20 dB HL or better at octave frequencies from 125 Hz to 8 kHz), normal middle ear function [within normal limits on acoustic immittance testing (ASHA, 1990; Roup, Wiley, Safady & Stoppenbach, 1998) and no self-reported history of factors that could affect their hearing ability when listening to the recording of the newly developed word list. They were considered to represent the general population in KZN, with participants coming from the following occupational groups: domestic worker, linguist, student, teacher, technician, librarian, clerk, factory worker and administrator.

### Materials and Instrumentation

#### The test word recording

The test word recordings used in this study were the 28 disyllabic isiZulu low-tone verbs recordings, selected during previous research for the development of an SRT test for isiZulu speakers in KZN, South Africa (Panday et al., 2007, 2009). This previous research had shown that these 28 word recordings satisfied the criteria of linguistic familiarity and homogeneity of audibility for the target population of isiZulu-speaking adults (Panday et al., 2007, 2009). The criterion of linguistic familiarity had been shown by having two Zulu speaking language interpreters and two tertiary-level educators identify 131 commonly used Zulu words, with 82% of these words subsequently identified as being disyllabic verbs. Five linguistic experts then rated 58 of these disyllabic verbs as being sufficiently familiar, phonetically dissimilar and low in tone to be potentially suitable for use in the development of an SRT test in isiZulu (Panday et al., 2007). The criterion of homogeneity of audibility had been shown by recording these previously identified 58 words from a male first-language speaker of isiZulu and playing these word recordings at six intensity levels to 30 isiZulu first-language speaking adults (aged 18 to 25 years) with hearing within normal limits (Panday et al., 2009). Logistic regression analysis was used to determine the psychometric function of each of the 58 word recordings, with 28 word recordings meeting the criterion of having a mean slope at 50% intelligibility within 1 SD of the group mean of 5.98%/dB. The prosodic features of these 28 word recordings were then analysed and their pitch contours were shown to conform to the prosodic pattern apparent within isiZulu linguistic structure (Panday et al., 2009).

**FIGURE 1:**
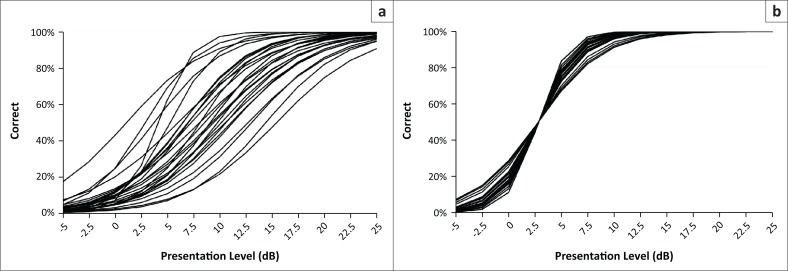
Psychometric functions for the 28 recorded isiZulu words unadjusted (a) and adjusted (b) for listener hearing thresholds.

Table 1 shows the 28 disyllabic isiZulu low-tone verbs used in this study and the psychometric properties of each word reported in previous research (Panday et al., 2009). For this study, the levels of each of the 28 word recordings were adjusted (ΔdB) so that the 50% correct perception scores for each word occurred at the mean pure-tone average (2.8 dB HL) of the participants in the previous homogeneity of audibility study (Panday et al., 2009). The size of this adjustment for each word is shown by the ΔdB levels in Table 1. Figure 1 shows each recorded word’s psychometric function before and after this level adjustment.

**TABLE 1:** Psychometric properties of the 28 disyllabic isiZulu word recordings used in this study.

Word	Slope at 50%	Slope from 20% to 80%	Threshold^a^	ΔdB^b^
qonda^c^	15.43	9.87	4.18	1.38
Thola	10.98	7.02	5.27	2.47
washa	9.58	6.13	2.90	0.10
thenga	8.83	5.65	8.13	5.33
Jeza	8.05	5.15	9.13	6.33
Yona	8.05	5.15	9.13	6.33
xola^c^	8.03	5.14	7.05	4.25
Yeka	7.90	5.06	6.52	3.72
Chela	7.68	4.91	9.82	7.02
Shada	7.60	4.86	6.51	3.71
gxeka^c^	7.50	4.80	3.74	0.94
Khaba	7.20	4.61	6.99	4.19
Veza	6.83	4.37	14.44	11.64
Thela	6.78	4.34	10.62	7.82
Yonga	6.53	4.18	7.31	4.51
Kheta	6.50	4.16	8.57	5.77
Wina	6.50	4.16	13.08	10.28
cinga^c^	6.45	4.13	11.28	8.48
Faka	6.43	4.11	1.10	3.63
Khipa	6.35	4.06	10.16	7.36
Thatha	6.05	3.87	11.11	8.31
Linda	6.00	3.84	9.45	6.65
Loya	5.93	3.79	12.73	9.93
minya	5.93	3.79	15.45	12.65
donsa	5.85	3.74	9.25	6.45
khanya	5.73	3.66	6.05	3.25
geza	5.70	3.65	8.11	5.31
banga	5.68	3.63	10.54	7.74

a , Mean presentation level (dB HL) required for 50% correct perception;

b , change in mean presentation level (dB) required to adjust the 50% correct perception threshold of a word to the mean three-frequency (0.5 kHz, 1 kHz and 2 kHz) pure-tone average (2.8 dB HL) of all participants (all changes required a decrease in level by the indicated amounts);

c , words containing click sounds.

#### The rating scale

The rating scale used in this study was developed based on previous research (Theunissen et al., 2009). It consisted of five questions asking the participant to rate each word on five criteria: pitch or tone, clarity/articulation, naturalness or dialect, speech rate and quality. Participants responded to each question on a 5-point Likert-like scale where 1 corresponded to strongly agree, 2 to agree, 3 to neutral, 4 to disagree and 5 to strongly disagree.

#### Data collection procedure

A pilot study was first conducted on three participants who were not part of the main study. The rating scale and test procedure were piloted for accuracy and ambiguity. Minor editorial and rephrasing of instructions were made. Thereafter, participants for the main study were recruited.

Each first-language isiZulu-speaking participant in the main study was shown the rating scale and instructed on its use. Each participant then listened to each of the 28 recorded isiZulu word recordings. Each word recording was played three times at a comfortable listening level of 60 dB HL, before each participant was asked to complete his or her ratings of that word recording. The word recordings were routed from a Technics (SLPG390) CD player to the external input of a Grason Stadler model 61 audiometer (calibrated in accordance with ANSI S3.6 specifications [ANSI, 2004]) and then to the test ear (chosen by flipping a coin) of each participant through a single TDH-49 headphone with MX41/AR cushions. Prior to testing each participant, the external inputs to the audiometer were calibrated to 0 VU using the 1000 Hz calibration tone on track one of the test CD.

#### Data analysis

The ratings, obtained from the 11 first-language isiZulu-speaking participants about the pitch or tone, clarity/articulation, naturalness or dialect, speech rate and quality of speech for each of the 28 isiZulu word recordings, were analysed descriptively and inferentially. Agreement amongst the 11 raters was calculated using percentage agreement. As these percentages were exceptionally high, further analysis using kappa statistics was abandoned in favour of Friedman analysis of variance (ANOVA) analyses (McHugh, 2012). These Friedman ANOVA analyses considered rater as the independent variable and the ratings in each rating category as the dependent variable. A Friedman ANOVA analysis was conducted for each rating category separately (pitch or tone, clarity/articulation, etc.).

All statistical analyses in this study were carried out using Stata version 14 under the guidance of a statistician from the Medical Research Council of South Africa (Durban).

## Ethical consideration

Unconditional ethical clearance was obtained from the University of Cape Town (HREC 652/2012). Verbal and written informed consent was obtained from all participants. All participants were given an information document that outlined the details of the study. Participants were assured of their anonymity and were free to withdraw from the study at any point. Confidentiality was maintained throughout the study.

## Results

**FIGURE 2:**
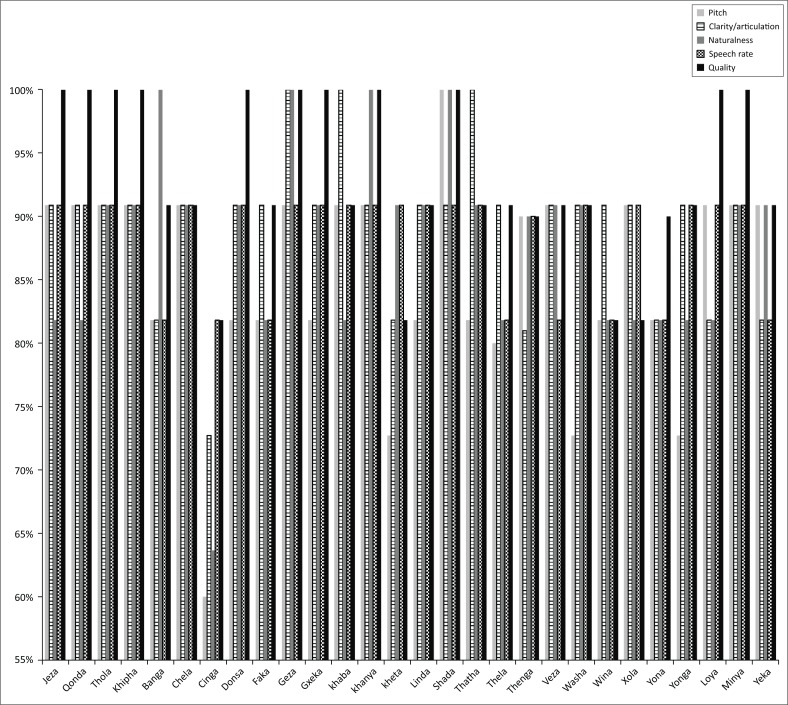
Percentage of raters strongly agreeing in each rating category for each of the 28 recorded isiZulu words.

Figure 2 shows ≥80% of the 11 first-language isiZulu-speaking participants rated 24 of the 28 isiZulu word recordings as ‘strongly agree’ in the categories of pitch or tone, clarity/articulation, naturalness or dialect, speech rate and quality. More than 20% of these 11 participants rated the three-word recordings – ‘kheta’, ‘washa’ and ‘yonga’ – below ‘strongly agree’ in the category of pitch or tone, and one-word recording – ‘cinga’ – below ‘strongly agree’ in the categories of pitch or tone, clarity or articulation and naturalness or dialect.

The assessment of rater reliability showed that the chi-square statistic of 18.31 (df = 10) was far greater than the calculated Friedman statistic in each rating category (pitch or tone, clarity/articulation, etc.) (Table 2). This led to the null hypothesis that there was agreement amongst the 11 participating raters within each of the five categories at a 95% confidence level.

**TABLE 2:** Friedman analysis of variance analyses comparing raters within each rating category.

Category	Friedman statistic	*p*
Pitch or tone	5.16	0.9
Clarity/articulation	3.05	0.9
Naturalness or dialect	4.73	0.9
Speech rate	2.07	0.9
Quality	3.44	0.9

## Discussion

The first-language speaker ratings were a valuable addition to the process of validating word recordings for use in a new SRT test. This was evident in at least four aspects of the rating scale: (1) By keeping the scale simple, it proved to be easy to develop; (2) By targeting the population for whom the SRT test was being developed, the participants proved to be easy to recruit; (3) With points one and two in place, the participants found the scale easy to complete; and (4) The results obtained from the scale were easily integrated with the linguistic and psychometric results already obtained for the SRT in its development to date and added evidence for the content validity of this test.

The first-language speaker ratings were also a useful addition to the process of validating word recordings for use in a new SRT test. This was most evident in all 28 word recordings having been previously accepted on linguistic and psychometric criteria (Panday et al., 2007, 2009), but the first-language speakers then identified potential problems with three-word recordings on pitch or tone and with one-word recording on pitch or tone, clarity or articulation and naturalness or dialect. A possible cause for these problems could lie in the manner in which the first-language isiZulu speaker spoke these words during the original recording. On reviewing these recordings, this speaker was heard to have provided greater emphasis on the second syllable of these words (an iambic meter). This resulted in a slight change in the pitch and tone of these word recordings, particularly for the word ‘cinga’. It was noted that this iambic meter (greater emphasis on the second syllable) differs from the spondaic meter (equal emphasis on each syllable) present in the disyllabic word recordings used in English language speech reception tests.

The value added by including first-language speaker ratings in the development of the isiZulu SRT test, considered in this study, supports suggestions that commonly used measures of linguistic criteria and homogeneity of audibility alone may not be sufficient to ensure that the final SRT test adequately represents all facets of the construct it has been designed to measure (Maxwell & Satake, 2006; Nissen et al., 2005). Considering broader criteria such as the appropriateness of the word recordings, as rated by members of the communities in which the SRT test will be used, can provide immediate and direct feedback as to the appropriateness of the proposed word recordings being trialled. Such ratings will contribute to the content and ecological validity of any final SRT test recording. Pascoe, Rogers and Norman (2013) indicate that there is need for researchers in South Africa to share the methods used to develop and validate contextually relevant tests. The use of community members or first-language speakers as experts of the language when selecting word recordings for SRT tests, is another method to consider when developing contextually relevant materials in South Africa.

## Conclusion

The first-language speaker ratings proved to be a valuable addition to the process of selecting word recordings for use in a new SRT test. In particular, these ratings identified potentially problematic word recordings in the new SRT test that had been missed by the previously, and more commonly, used linguistic and psychometric selection criteria.

This study has implications for the methods used to determine the reliability and validity of new tests for speech audiometry. It supports the use of multiple methods to systematically accumulate evidence for and against the use of new tests, and the viewing of this evidence in an integrated way to determine the true reliability and validity of new tests in the settings in which they will be used.
